# Discovery of Protein Phosphorylation Motifs through Exploratory Data Analysis

**DOI:** 10.1371/journal.pone.0020025

**Published:** 2011-05-25

**Authors:** Yi-Cheng Chen, Kripamoy Aguan, Chu-Wen Yang, Yao-Tsung Wang, Nikhil R. Pal, I-Fang Chung

**Affiliations:** 1 Institute of Biomedical Informatics, National Yang-Ming University, Taipei, Taiwan; 2 Center for Systems and Synthetic Biology, National Yang-Ming University, Taipei, Taiwan; 3 Department of Biotechnology and Bioinformatics, North Eastern Hill University, Shillong, India; 4 Department of Microbiology, Soochow University, Taipei, Taiwan; 5 Software Technology Division, National Center for High-Performance Computing, Taichung, Taiwan; 6 Electronics and Communication Sciences Unit, Indian Statistical Institute, Calcutta, India; Semmelweis University, Hungary

## Abstract

**Background:**

The need for efficient algorithms to uncover biologically relevant phosphorylation motifs has become very important with rapid expansion of the proteomic sequence database along with a plethora of new information on phosphorylation sites. Here we present a novel unsupervised method, called Motif Finder (in short, F-Motif) for identification of phosphorylation motifs. F-Motif uses clustering of sequence information represented by numerical features that exploit the statistical information hidden in some foreground data. Furthermore, these identified motifs are then filtered to find “actual” motifs with statistically significant motif scores.

**Results and Discussion:**

We have applied F-Motif to several new and existing data sets and compared its performance with two well known state-of-the-art methods. In almost all cases F-Motif could identify all statistically significant motifs extracted by the state-of-the-art methods. More importantly, in addition to this, F-Motif uncovers several novel motifs. We have demonstrated using clues from the literature that most of these new motifs discovered by F-Motif are indeed novel. We have also found some interesting phenomena. For example, for CK2 kinase, the conserved sites appear only on the right side of S. However, for CDK kinase, the adjacent site on the right of S is conserved with residue P. In addition, three different encoding methods, including a novel position contrast matrix (PCM) and the simplest binary coding, are used and the ability of F-motif to discover motifs remains quite robust with respect to encoding schemes.

**Conclusions:**

An iterative algorithm proposed here uses exploratory data analysis to discover motifs from phosphorylated data. The effectiveness of F-Motif has been demonstrated using several real data sets as well as using a synthetic data set. The method is quite general in nature and can be used to find other types of motifs also. We have also provided a server for F-Motif at http://f-motif.classcloud.org/, http://bio.classcloud.org/f-motif/ or http://ymu.classcloud.org/f-motif/.

## Introduction

Protein phosphorylation, mediated via a group of enzymes (called kinases) that performs addition of a phosphate (PO4) group usually to serine (S), threonine (T), tyrosine (Y) residues, is one of the most frequent forms of post-translational modification mechanisms. In prokaryote, aspartic acid/glutamic acid might be considered to be involved in the process through histidine-specific protein kinases. Although only 518 kinases are identified among about 30,000 human proteins, more than 30% of human proteins are affected by kinase-mediated phosphorylation and nearly half of kinases (244) have close relationships with cancers and other diseases [1], [2]. Modification of proteins via phosphorylation is considered a key event that is involved in the most abundant form of cellular regulation including metabolism, signal transduction pathways, transcription, translation, membrane transport, cell growth and cell differentiation [3], [4]. Thus, gaining an understanding of the mechanism of kinase-specific phosphorylation is an important step in explaining the role of protein functions in the regulation of cellular processes.

Recently, through *in vivo* or *in vitro* experiments and due to the advent of mass spectrometry techniques, a huge number of phosphorylation sites have been identified and collected in a number of databases, such as “PHOSIDA” [5], “Phospho.ELM” [6], and “PhosphoSitePlus” (http://www.phosphosite.org/). Furthermore, patterns (or motifs) surrounding phosphorylation sites, which are viewed as the guidance rules for different kinases to recognize their corresponding protein substrates, have also been gathered in several databases, e.g., “PhosphoSitePlus”, “Scansite” [7], “Mini-motif Miner” [8], and “PhosphoMotif Finder” [9]. However, even though a huge amount of phosphor-proteomics data across a wide range of species are being continuously generated [10], [11], [12], information about the kinases responsible for such modification is lacking. Hence, many computational methods have been developed, based on the assumption that each kinase recognizes its corresponding specific substrate peptides in ensuring phosphorylation signaling fidelity, to identify the plausible kinase. These methods can provide fast and automatic annotation of kinase-specific phosphorylation. In this context, investigators have focused attention on two kinds of *in silico* research topics: phosphorylation site prediction [13], [14], [15] and phosphorylation motif extraction [16], [17].

Considering the fixed length of aligned peptides centered on serine residue, here we focus on extraction of a set of phosphorylation motifs that are over-represented in the phosphorylated peptides but under-represented in the unphosphorylated peptides. A similar strategy is also adopted in other studies [16], [17]. Such approaches can help us to extract consensus motifs hidden in substrate peptides surrounding phosphorylation sites. These motifs may provide more precise rules to guide kinase-substrate recognition. In a previous study [18], phosphorylated peptides are further clustered into several subgroups to extract more delicate patterns for increasing the accuracy of phosphorylation site prediction. There are several other protein/DNA motif extraction methods that can be used to analyze phosphorylation data [19], [20], [21]. These methods are based on groups of long *unaligned background* sequences to score motifs, a concept motivated by the discovery of transcription factor binding sites. Since these methods consider mixed information surrounding phosphorylated and unphosphorylated sites, they do not explicitly identify motifs in a set of phosphorylated peptides.

As mentioned earlier, using a set of aligned phosphoproteomics data, two methods, called Motif-X [16] and MoDL [17], were developed for identifying phosphorylation motifs. In fact, these two methods identify two different kinds of motifs. Motif-X tries to extract a type of motif with only one identifiable character in a specific locus (For example, a 13-mer pattern = “…RR.S……”). MoDL identifies motifs with a *mixture* of information (more than one possible character) in a specific position (ex., pattern = “…[RK][RTD].S……”). Here “…” represents wild-card characters (any character is possible). These two types of motifs are quite popular and useful to biologists to examine consensus information in given sequences. In this study, we mainly compare the performance of our method with that of Motif-X because our new scheme extracts motifs of the same type found by Motif-X. Our method usually finds the same set of motifs found by Motif-X, but often it also finds delicate patterns overlooked by Motif-X as a consequence of the fact that Motif-X extracts patterns initially using *all* of a given set of phosphorylated peptides. By using progressive clustering of the data, we have been able to present an improved approach that can detect those key delicate patterns overlooked by Motif-X.

We have also made a comparison of our method with MoDL. However, the motifs sought by MoDL are mixture motifs and therefore not strictly the same as those sought by our method. Thus to make a proper comparison, we break up a mixture motif found by MoDL into motifs with single residues (e.g., [RK][RK].S can be thought of as RR.S, RK.S, KR.S and KK.S), and then check the statistical significance of these single-residue motifs. We have found that our method finds those motifs which are extracted by MoDL and are statistically significant. Some of the motifs found by MoDL are statistically not significant and our method does not identify them, thereby providing an important improvement. Moreover, our method also discovers some novel motifs not identified by MoDL. Since we compare our results with Motif-X and MoDL, for the sake of completeness we provide a very brief introduction of them in Text S1.

**Figure 1:**
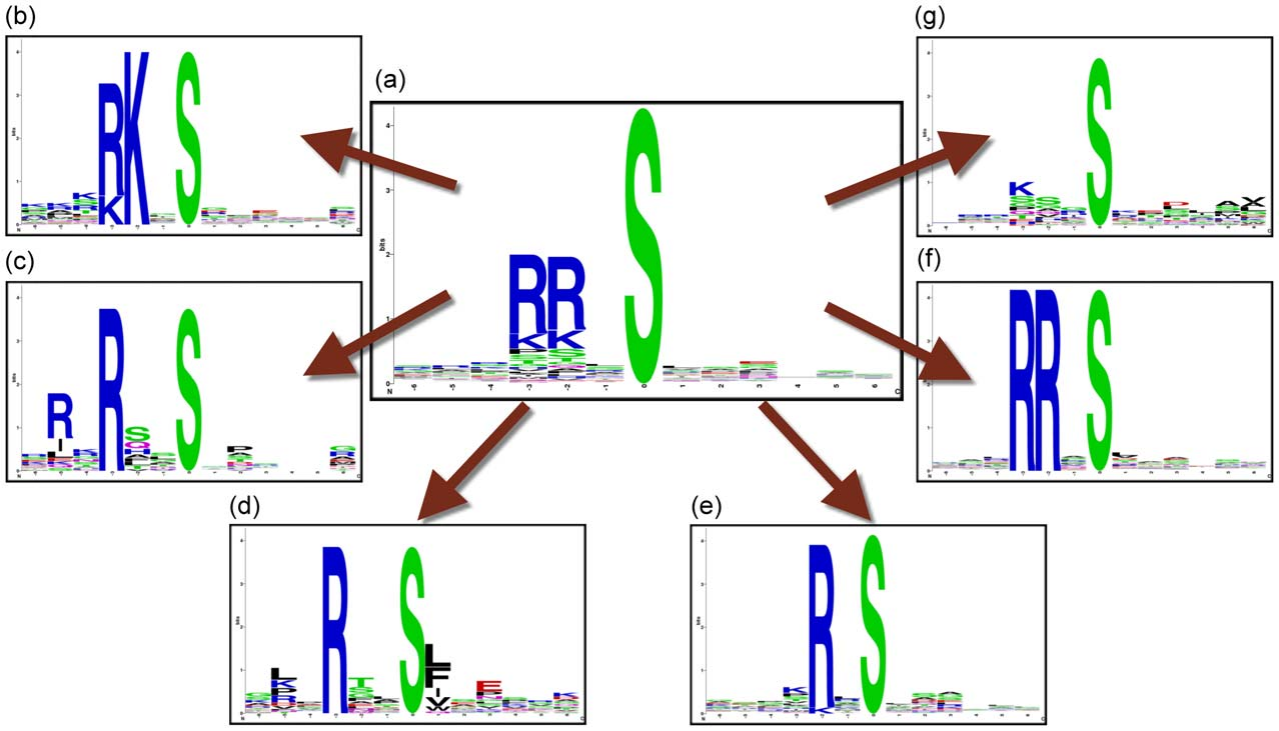
An illustration of extraction of different consensus sequence motifs by clustering process. A set of fixed length sequences are represented by a sequence logo. Sequence logo (a) represents all of the PKA kinase substrates. The sequences in (a) are split into several clusters. Each cluster is then represented by a sequence logo. Sequence logos (b)∼(g) represent PKA kinase substrates in different clusters.

Most motif finding methods usually try to extract patterns initially using all of a given set of phosphorylated peptides. However, these approaches often result in failure to identify delicate patterns that are obscured by stronger patterns in the data. Since we believe that some key patterns can *only* be observed in a *part* of a set of phosphorylated peptides, we use progressive clustering of the data, which results in a refined approach, to identify the key patterns. Clustering techniques can be very effective in grouping peptides with highly similar patterns into clusters. Thus, we may be able to extract consensus sequence motifs from those clusters. We briefly illustrate this process in Figure 1. Considering a set of PKA kinase substrate peptides, the subfigure 1(a) at the center depicts the sequence logo of the PKA kinase foreground data. After clustering this foreground data, we can get the subfigures 1(b)∼(g) representing various motifs: “….R.S……”, “…R‥S……”, “…RK.S……”, and “.R.R‥S……”, etc. Thus, we see that each such cluster is rich enough in information to extract the kinds of motifs identified by both Motif-X and MoDL. In this study we propose an algorithm, called F-Motif, that not only extracts motifs of the type identified by Motif-X, but usually also outperforms the latter in several respects, including finding additional motifs not found by Motif-X. Facts from published literature, reveal that some of these additional motifs (found by F-Motif) are well established using laboratory experiments. Further, F-Motif also uncovers novel substrate motifs that are yet to be assigned to specific kinases. Also we have checked the statistical significance of each of the single-residue motifs obtained after breaking up mixture motifs identified by MoDL. We find that F-Motif identifies all of the statistically significant motifs identified by MoDL, and ignores motifs found by MoDL which are not statistically significant. F-Motif also identifies motifs that are novel and not identified by MoDL.

## Materials and Methods

### Data Sets

In this study we have performed four sets of experiments to compare the motif-discovery ability of F-Motif with that of Motif-X and MoDL. In one of our experiments, as our foreground data we have collected only the serine-phosphorylated peptides of length 13 (i.e., 13-mers centered at serine) from the Phospho.ELM database (Version 8.1) [6], which are experimentally determined to be substrates of different kinases. Also, considering all available proteins in the Phospho.ELM database, we have extracted all peptides of length 13, (i.e., 13-mers) centered on serine as one kind of background data. Thus this background data set consisting of more than 300,000 peptides includes both serine-phosphorylated and serine-non-phosphorylated peptides. We shall denote the foreground data set as *F* while the background data set will be called *B*. Based on the species information available for those proteins in the Phospho.ELM database, we have further separated this background data into two species-related background data sets: considering only human species (more than 200,000 peptides) and all species together. Considering the interactions of phosphorylated peptides with different kinases and species, several foreground data sets are created. More specifically, four foreground data sets are generated for human species, one for each of the four kinases, PKA, PKC, CK2, and CDK. Similarly, we have created four other foreground data sets considering all species together.

For comparison, we shall also use two foreground data sets from Schwartz and Gygi [16] in conjunction with different background data sets generated from the Phospho.ELM database. In [16], one foreground data set collected by Schwartz and Gygi comprised multiple sets of different kinase substrates, and the other one was artificially generated by them containing five synthetic motifs. Finally, we shall use a large-scale mass spectrometry data set for mouse species with more than five thousand serine-phosphorylated sites [22]. Note that, in our experimental analysis with the mass spectrometry data for mice, we use a background data set different from the first three experiments. This background data set is obtained from the IPI mouse database (http://www.ebi.ac.uk/IPI/) also considering peptides from mouse proteins of length 13-mers centered at serine. For a fair comparison with Motif-X and MoDL, we have removed multiple copies of same 13-mers (since both methods removed multiple copies). However, if the multiple copies of a 13-mers are coming from diffe`rent proteins, it may not be rational to remove them. For Experiment 2 (described later), as an illustration, we have reported the additional motifs that F-Motif can get without removing the additional copies of repeated 13-mers. These foreground and background data sets are briefly summarized in Table 1 and further explained in Text S1.

**Table 1:** Summary of the data sets.

	Data set (No. of peptides)	Description
Foreground data sets	*FM* (298)	Foreground data set comprised of the multiple sets of ATM, Casein II, CaMK II, and MAPK kinase substrates [16].
	*FA_PKA_* (306)	Foreground data sets from the Phospho.ELM database considering all species with respect to PKA, PKC, CK2, and CDK kinase substrates.
	*FA_PKC_* (297)	
	*FA_CK2_* (241)	
	*FA_CDK_* (209)	
	*FH_KA_* (187)	Foreground data sets from the Phospho.ELM database considering only human species with respect to PKA, PKC, CK2, and CDK kinase substrates.
	*FH_PKC_* (209)	
	*FH_CK2_* (177)	
	*FH_CDK_* (155)	
	*FS* (9774)	Synthetic foreground data set consisting of five specially designed motifs “…D‥SQ.N…”, “….R.S‥L…”, “…TV.S.E….”, “….R.S‥P…”, and “…‥KS…I‥” [16].
	*FMS* (4189)	Foreground data set from mouse mass spectrometry data [22].
Background data sets	*BA* (346248)	All species background data set from the Phospho.ELM database.
	*BH* (233805)	Human background data set from the Phospho.ELM database.
	*BM* (514792)	Mouse background data set from the IPI mouse database.

### Motif Finding Algorithm

A sequence motif is a pattern that appears frequently in a group of related proteins or DNA sequences and may correlate with some biological functions [23], [24]. Here we are interested to find motifs in phosphorylated proteins. To identify such motifs *algorithmically* we need some *computational definition* for a motif. Different researchers use different definitions for this [16], [17]. In order to find a motif, Motif-X [16] uses two natural conditions: each residue/position pair in a motif should be statistically significant and a motif must appear in the foreground data adequately. To satisfy the first condition the *P*-value for each residue/position pair in a motif, computed according to a Binomial distribution based model, should be less than a threshold (*P_T_*) and to satisfy the second condition the frequency of occurrence of a motif in the (current) foreground data should be more than a threshold (*M*). *In this study we use this definition to discover motifs.* Clearly, different choices of *P_T_* and *M* may result in different sets of motifs and we cannot make inferences about false positive/negative without wet-lab validation. If we use a stricter threshold for *P_T_* and *M*, the identified motifs are more likely to be true motifs, but we may also miss some true motifs as well. Since such computational methods do not explicitly use any biological knowledge, such situations will arise with *all* computational approaches. We emphasize that *P_T_* and *M* are not algorithmic parameters but are related to the *definition* of a motif. *In Motif-X, M = 20 and P_T_ = 10^−6^ have been used. So, to compare our results we also use the same protocol.*

**Figure 2:**
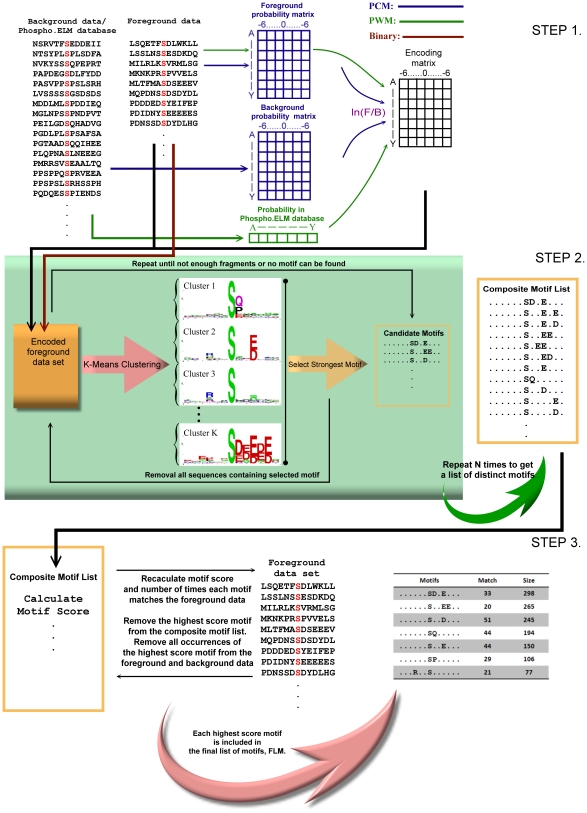
Overview of motif finding steps. In Step 1, for PCM we use the background data and foreground data, for PWM encoding, in place of the background data we use the entire Phospho.ELM database, while for binary encoding neither the foreground nor the background data are used. In Step 2 the *k*-means clustering algorithm is repeatedly used to generate a composite motif list (*CML*). This *CML* is then used to generate the final list of motifs in a stepwise manner ensuring two factors: statistical significance of the motif using a Binomial distribution based model, and frequency of occurrence of the motif in the present foreground data is at least *M*.

Let *F* be the original sequence data consisting of a set of sequences of length 13-mers of serine-phosphorylated peptides. To find the motifs we cluster these serine-phosphorylated peptide sequences, because a motif represents highly conserved positions and we expect to have clusters representing instances that match a particular motif. In other words, if we cluster sequences of phosphorylated peptides, subsets of sequences with the same conserved positions should be part of the same cluster. To cluster the peptide sequences, we use an appropriate encoding of the sequences to get a numeric representation, F*, of *F*. In this study we use the *k*-means algorithm (explained in Text S1), but other clustering algorithms such as the Fuzzy *c*-means may also be used [25]. Once we get such clusters, we can analyze them and identify the motifs. This is our strategy here. To implement such an approach, we use a three-step procedure as outlined in Fig. 2: Encoding of sequence data, Clustering and finding of potential and candidate motifs, and Identification of the motif set.

#### Step 1: Encoding of sequence data

For useful clustering of sequence data the choice of the encoding method, to convert *F* to F*, is of utmost importance. The success of such a method may significantly depend on the encoding method used. Here we use two kinds of score matrices for encoding: Position Contrast Matrix (PCM) and Position Weight Matrix (PWM) (explained below). PWM has been used in other studies but for different problems [26], [27]. In addition to these, we also explore the effectiveness of binary coding (to be explained later). The encoding method should enhance the contrast between the foreground data (sequences of phosphorylated peptides) and background data (sequences of phosphorylated and non-phosphorylated peptides). The score matrices are defined using some foreground (*F*) and some background (*B*) data sets. The encoded foreground data set is represented as F*.

- **Position Weight Matrix (PWM)** [**28]**, [29****] **.** This score matrix is defined primarily using the foreground data. We calculate the relative frequency of a residue at each position in the foreground data and divide it by the relative frequency of that residue in the entire Phospho.ELM database. The relative frequency of residue *i* at location *j* is computed as 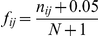, where 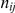 is the frequency of residue *i* at location *j* in the foreground data and 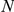 is the number of fragments in the foreground data (i.e., the size of the foreground data set). The addition of 0.05 is to ensure that 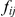 never becomes zero as we have to take the logarithm of it. The constant 1 in the denominator can be dropped, but we have kept it as other researchers have used it [29], [30]. Let 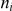 be the frequency of residue *i* in the entire Phosph.ELM database and 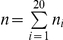 and then the relative frequency of residue *i* is given by 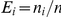. Let us illustrate it using a fixed length (2*s*+1)-mers, where *s*>1 is an integer. For example with *s* = 6, we have fragments of length 13. Thus, for *s* = 6, the PWM is defined as
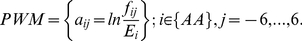
(1)Here 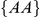 is the set of residues.
- **Position Contrast Matrix (PCM).** An effective encoding scheme should enhance the contrast between the foreground and background data. Thus, the Position Contrast Matrix is defined using the ratio of probabilities of a residue at a particular position in the foreground data and in the background data. Thus the PCM score matrix for fragments of length 13-mers (i.e., *s* = 6) is defined as
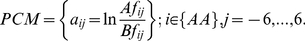
(2)In Equation (2) 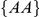 is the set of residues, 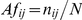 is the probability of occurrence of residue *i* at position *j* in the foreground data, while 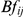 is the same in the background data. Note that, 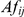 is essentially the same 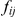 used in Equation (1). Given a sequence of residues of length 13, we replace the residue *i* at position *j* by 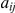 resulting in a vector of length 13 as explained in Figure 2.

Note that, we can use any other meaningful coding scheme including binary coding. The coding scheme should enhance the distinction between the phosphorylated and background sequences. We shall demonstrate later that binary coding is also reasonably effective with our algorithm.

#### Step 2: Clustering and finding of potential and candidate motifs

The general outline of the Step 2 process is to cluster the phosphorylated data F* into *k* clusters. Then from each cluster we select one potential motif, if that satisfies our criteria. Thus from *k* clusters we can get a list of at most *k* potential motifs. From this list of potential motifs, we select the best one (please refer to the filtering rules described later in Task-2). This best potential motif is added to a *list of candidate motifs*, called “*CM*”, *CM* = {*m_1_*, …, *m_t_*}; *t* is the number of distinct candidate motifs. This process of finding one candidate motif is called a *trial*. After this, all occurrences of this best motif are removed from phosphorylated data *F* and F* and the clustering of the reduced set of phosphorylated data is done to find the next best candidate motif. This process is repeated till no more candidate motif can be found. This entire process will be called an *iteration*. Thus, Step 2 is a process of repeated cycles (iterations) designed to identify potential and candidate motifs. Each iteration comprises of trials, and each trial consists of two tasks (described later in detail).

Since the clusters found by the *k*-means algorithm depend on the initialization used, we repeat the iterations several times (in this case 50 times) and each iteration may result in a different list of candidate motifs. In other words, different iterations may result in different sets of motifs. However, highly conserved regions, i.e., strong motifs are expected to appear repeatedly in different iterations. We record the candidate motifs from all iterations to form *the composite list of motifs* and that list is denoted by *CML* = {*m_1_*, …,*m_T_* }; *T* is the number of distinct candidate motifs.

While forming the list of candidate motifs, two questions arise (i) Should every best motif from a cluster be considered a candidate motif? (ii) How many clusters should be looked for? The two questions are related and we shall address them together. The answer to the first question is, “No”. The best motif must have adequate representation in the foreground data set. Let *N* be the size of the foreground data set F*. Following the same protocol as that of Schwartz and Gygi [16], we assume that for a candidate motif, its frequency of appearance in *F* should be at least 20. Here let this minimum required frequency be *M*. We want to partition F* into *k* clusters with a hope that each cluster may represent one or more motifs. In order to get a motif with a frequency of appearance at least *M*, each cluster size should *preferably* be greater than *M* because every member in a cluster may (usually *will*) not satisfy the motif. We use the word *preferably* because although most data points satisfying the motif are expected to be in the cluster, there may be (usually *will* be) some fragments outside the cluster that also satisfy the motif. We assume that to get a motif, on average a cluster size should be 1.5*M*. In the absence of any information, we may assume that clusters will be of uniform size (a kind of the maximum entropy choice), thus the desired value for the number of clusters is *k* = *N*/(1.5*M*). However, as we continue finding motifs, the size of the data set F* will reduce and hence *k* will also change. At any stage of clustering, if we get some clusters with very small sizes, then also the number of clusters will be reduced. We shall explain this later in detail.

In our implementation, to be consistent with Schwartz and Gygi [16], we have used *M* = 20 and hence we start with *k* = *N*/30 clusters. Note that, one can use some cluster validity measures [31], [32] but we do not adopt such a path for two reasons: First, there is no universally acceptable cluster validity index. Second, in the present case, our intention is not to look for clusters in the pattern recognition sense but to use it as an aid to find motifs.

**Figure 3:**
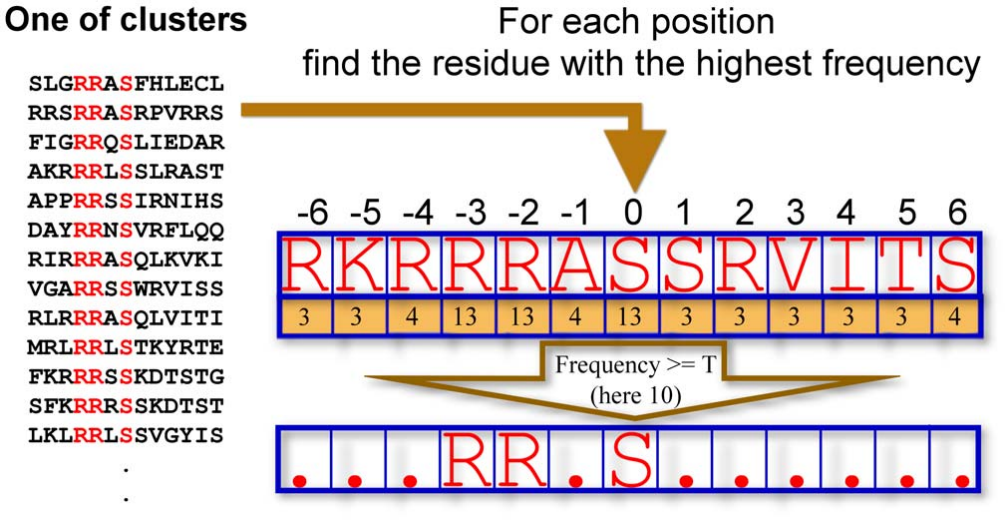
An illustration of how a potential motif is extracted from a cluster. First, for every position the frequency of each residue is counted. Then for each position the residue with the highest frequency is noted. If more than one residue have the same highest frequency, one of them is randomly chosen. At the next stage of the process, sites with residues having frequency ≥*T* are considered conserved sites to generate a potential motif.

- **Task-1: Identifying Potential Motifs from Each Cluster.** So far we have not explained how to identify potential motifs. As mentioned before, in each trial, we first generate a set of *k* = *N/30* clusters *C_1_*, *…*, *C_k_*. If a cluster is very small, it is not likely to yield any motif. Hence any cluster with a size smaller than a threshold *G* is treated as a small cluster and discarded (we discard the cluster but not the points in the cluster), and the task of identifying potential motifs is continued with a reduced number of clusters, as elaborated later. The data points in each cluster are expected to be homogeneous (similar) and hence may have some motifs. For each cluster, we calculate the frequency of each amino acid at each position. Let the frequency of *AA_i_* at position *j* in a cluster be *r_ij_*. In a cluster, if the frequency of a particular *AA* at one position is large enough, then that position may be a conserved position and hence a possible candidate for a motif. If *r_ij_* is greater than a threshold *T*, then the *j* ^th^ site may be conserved with residue *i*. But there may be more than one such residue for the same position. So we find the residue with the highest frequency for position *j* and take that as conserved. For a given *j*, if more than one residue have the same highest frequency then only one of them is selected randomly.Since *M* is the minimum required number of occurrences of a template to be considered a motif, *T* should be ≤*M*. Similarly, *G* should also be ≤*M*. But the use of a *T* that is too small is not desirable. Similarly, a very small *G* is also not desirable. When we use, say, *M* = 20 and *T* = 20, one may think, that *G*>20 would be desirable. This is not so, because for a given motif, all fragments satisfying that motif may not (usually will not) appear in the same cluster. Hence, in our all experiments, we have used *G* = 15 and *T* = 15 as default values, although we have studied separately the robustness of the algorithm with respect to various other choices of *G* and *T*. In this way, from each cluster we shall identify only a very strong potential motif out of the possible candidates, noting however the possibility, that a cluster may yield no potential motif. Figure 3, illustrates this process of finding a potential motif from a cluster.
- **Task-2: Filtering of the Potential Motifs.** All potential motifs may not be good ones. In any trial, for each potential motif first we count the total number of peptides in *F* that satisfy the motif. If this count is not equal to or greater than *M* (here *M* = 20), we discard that potential motif. We use *M* = 20 because Schwartz and Gygi [16] used the same threshold. From an algorithm's point of view we assume that motifs with a larger number of conserved positions are better than those with a smaller number of conserved positions. Note that, we do not, in any way, like to suggest that longer motifs are biologically more important or more informative. With a view to finding longer motifs, in this reduced set of potential motifs we find the motif with the highest number of conserved positions. If there is just one motif, with the largest number of conserved sites, we pick it up as a candidate motif from this trial and add it to *CM*. If there are more than one motif with the same highest number of conserved positions, then we pick up the one with the smallest frequency of occurrence in the original *F*. We do so to enhance the possibility of finding additional motifs. If there is a tie in terms of frequency, we randomly pick any one of the tied potential motifs. Finally, before we finish the trial, all sequences in *F* that match the selected motif are removed from *F* to get the reduced *F* and F* that will be used in the next trial.
- **Iteration – Repeat the trials.** Now we repeat the trial with the reduced *F* and F* to find additional motifs. If in the most recent trial, there are *k>0* clusters and none of them result in a potential motif, then possibly we have over-clustered, i.e., generated more clusters than we should have. Hence, for the next trial we set *k = k−1* and repeat the process. Or, we shall find, *TSn*, the total number of fragments in all of the *Sn* small clusters (i.e., sum of the sizes of the *Sn* small clusters). Since these *TSn* fragments may generate 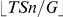 useful clusters, we reduce the number of clusters by 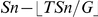 clusters. In other words, the next trial starts with 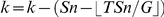.In this way, trials are repeated, i.e., Task-1 and Task-2 are repeated until *k* becomes zero. This finishes one motif-finding iteration. At the end of an iteration, the candidate motif list *CM* is copied to composite motif list *CML*.
- **Repeat the iterations.** Because the results of *k*-means clustering depend on the initialization, if we repeat the iteration described above, we are likely to get a set of motifs that may (usually will) not be identical to the list of motifs extracted in some other iteration. But, strong motifs are likely to be selected more frequently than weaker ones. Hence, we repeat the iteration *IT* ( = 50) times. Each time we get a list of motifs, which is copied into *CML*. We also compute the frequency with which different motifs are selected over iterations. This *CML* will now be further filtered, as discussed in Step 3 below, to identify *the final list of motifs*, *FLM*.

#### Step 3: Identification of the motif set

Thus far in the process, although we have tried to extract phosphorylation motifs that are over-represented in the phosphorylated peptides (foreground data), we have not yet established whether those motifs are in fact under-represented in the unphosphorylated peptides. Step 3 finds *the final list of motifs*, *FLM*, a set of reliable motifs (from *CML* produced in Step 2) that have met *both* criteria, over-representation in the phosphorylated peptides, and under-representation in the unphosphorylated peptides. In order to do that, as mentioned earlier, we follow Schwartz and Gygi [16] by considering two factors: (i) statistical significance of a motif using a Binomial distribution based model, and (ii) whether the frequency of occurrence of the motif in the current foreground data is at least *M* (here *M* = 20). To assess the statistical significance of a motif, we use the same criterion as used in [16]. The association between a position-residue pair is considered statistically significant, if the *P*-value of that pair smaller than a threshold (here the threshold is 10^−6^). Consequently, a motif is considered statistically significant, if each of the position-residue associations in the motif is statistically significant. Instead of using the *P*-values, as suggested in Schwartz and Gygi [16], the statistical significance of a motif can also be assessed using the motif score defined in Equation (3). The motif score is computed using a log transformation of the Binomial probabilities (e.g., *P*-value of 10^−6^ = motif score of 6):
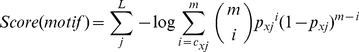
(3)where *m* is the size of *F*, 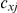 is the frequency (count) of residue 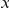 at position *j* in *F*, 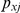 is the relative frequency (fractional percentage) of residue 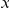 at position *j* in *B*, the background data, *L* is the length of the motifs, i.e., the number of conserved positions. If we use a higher threshold (say 10^−4^) on the *P*-value or if we do not impose the constraint on *P*-value then F-Motif may generate a bigger list of motifs. For example, we shall see in Experiment 2 that if we ignore the constraint on *P*-value, F-Motif identifies the motif “……SP.P…” with a high score of 21. But this motif is lost when we impose the constraint on *P*-value, because although the *P*-value associated with “……SP…‥” is about 10^−16^, the same for “……S‥P…” is about 10^−5^. For a fair comparison, following Schwartz and Gygi [16], when the motif score at any motif site is larger than 16, it is taken as 16.

- **Calculate motif score.** In this step, we use *F* and *B* to calculate the motif score for each candidate motif in *CML*.
- **Refine the motif set.** We select the motif in the *CML* that satisfies the constraint on the P-value and has the highest score, and add it to the final list of motifs, *FLM*. If there is more than one motif with the same highest score, we pick the one which has the most occurrences in *F*. If there is a tie in the number of occurrences in *F*, then any one of the tied motifs can be selected. Then we remove all sequences from *B* and *F* that satisfy the motif. Note that, before selecting a motif we must ensure that it appears at least *M* times in the present foreground data. Now using the reduced *F* and *B*, we re-estimate the probabilities used in Equation (3) and re-compute the scores of the remaining motifs. Based on these new probabilities and new motif scores, using the same procedure we select the next motif with the highest score and copy it to *FLM*. This is followed by removal of all occurrences of sequences from *B* and *F* that satisfy the motif. This process of selection is continued until no more statistically significant motif is found.

### Summary of F-Motif Algorithm

To summarize, the algorithm has two phases, one for finding a list of candidate motifs and the second for finding the motifs satisfying our definition. The first phase explores and identifies as many candidate motifs as possible using a clustering environment. This idea is different from other approaches and is the strong point of our philosophy because, unlike Motif-X, it allows us to find key subtle patterns by looking at *parts* of a set of phosphorylated peptides. For the second phase, we provide a scheme to find the final list of motifs, but note that it is possible to design other schemes.

### Algorithm F-Motif Pseudo-code

STEP 1.

  1. Get Foreground (*FG*) and background (*BG*) sequence data
  2. Encode the *FG* sequences

STEP 2.

  1. Repeat Iterations (*IT*) with *FG*
  2. Repeat Trials
  3. Compute the number of clusters, *c*
  4. Cluster current *FG*
  5. Generate potential motifs (at most one from each cluster) with the frequency in *FG*≥*M*
  6. Generate a candidate motif from the potential motifs having the highest number of conserved positions (in case of ties, select the one having the minimum frequency in *FG*)
  7. Remove all occurrences of the candidate motif from the current *FG*
  8. Until we fail to get a candidate motif
  9. Add candidate motifs to composite motif list (*CML*)
  10. Until *IT* times
  11. Compute frequency of every motif in *CML*

STEP 3.

  1. Generate final list of motifs (*FLM*)
  2. Compute Motif Score and check *P*-value for every motif in *CML*
  3. Select best motif in terms of score (in case of ties, select the one with maximum frequency in current *FG*) and its frequency in the current *FG* must be ≥*M*
  4. Remove the all occurrences of the selected motif from *FG* and *BG*
  5. Until no more acceptable motif is found

## Results

### Motif Discovery Results

In order to benchmark our method, primarily we have used a state-of-the-art method, Motif-X [16] for comparison. In addition, we have also compared our results with those from MoDL [17]. We shall denote our algorithm as F-Motif. For a fair comparison, we use the same parameters, to the extent possible, for both Motif-X and F-Motif. For example, we use 13-mers peptides for both algorithms. Also, as used in Schwartz and Gygi [16], in order to qualify as a motif, the minimum required frequency of a motif in the current foreground data is set to *M* (here *M* = 20). As mentioned in the Materials and Methods, we have four data sets: a set of data generated by us, two data sets obtained from Schwartz and Gygi [16], and another data set on mouse mass spectrometry [22]. For comparison, we use all three methods, Motif-X, MoDL, and F-Motif, on all four sets of data. Our method, F-Motif and Motif-X, both compute the motif score using Equation (3) as done in Schwartz and Gygi [16]. For a proper comparison with MoDL as mentioned in the Introduction, we break up a mixture motif found by MoDL into motifs with single residues, and then we analyze the statistical significance of those single-residue motifs. In order to show statistical significance of these single-residue motifs, we do not directly use Step 3 of our F-Motif algorithm. In Step 3, after we find a motif we remove occurrence of that motif from the foreground and background data sets. Thus the order, in which we consider these single-residue motifs, will have strong impact on the statistical significance, and some of these motifs are not likely to be significant. Hence, to favor MoDL, we individually check statistical information for these single-residue motifs and indicate whether each of the associations between the position-residue pair in each motif is statistically significant in terms of *P*-value.

#### Experiment 1: *FM* in conjunction with *BA* and *BH*

We have extensively studied the effect of choices of *G* and *T* (discussed in Text S1) on the performance of F-Motif and based on that we recommend the use of *G* = 15 and *T* = 15. Except the experiments that investigate the effect of choices of the two parameters, we use *G* = 15 and *T* = 15 as in Table 2. Table 2 summarizes the motifs obtained by Motif-X and F-Motif using the foreground data set, *FM* from Schwartz and Gygi [16] in conjunction with both background data sets, *BH* (only human species) and *BA* (all species). In column 3 and column 6 of Table 2, the pair of values (*a*, *b*) shows the motif scores: *a* is the score when *BH* is used and *b* is the score when *BA* is used. Note that, for the first detected motif (by both algorithms) the scores computed by Motif-X and F-Motif are different. This is probably because Motif-X uses the “pbinom” function of PERL while we make a logarithmic transformation of the terms in Equation (3) to compute its value. If a motif is found by both F-Motif and Motif-X, then in the column labeled “Index” we include the order in which that motif is detected by Motif-X. We follow this convention in subsequent tables also. The last column in Table 2, indicates whether these motifs also appear in the MoDL results when *BH* or *BA* is used (discussed in next paragraph) – a symbol “✓” indicates that the motif is found by MoDL while “×” indicates that it is not found by MoDL. Table 2 reveals that F-Motif finds all motifs identified by Motif-X. It also discovers a new motif “……S‥EE‥” shown in row 2 of Table 2. Since Motif-X cannot find this motif, we use an asterisk (*), to indicate that this is a new motif and *1 indicates that it is the first new motif identified by F-Motif. It is interesting to note that this motif has a higher score than five of the motifs found by both algorithms. Also, note that we perform an exhaustive search to uncover motifs of length up to four, and find that there are only those seven motifs satisfying the constraint on *P*-value. Later we shall discuss whether such new motifs are indeed novel.

**Table 2:** Motifs identified by F-Motif and Motif-X using the foreground data set, *FM* and the background data sets, *BH* and *BA* for *G* = 15 and *T* = 15.

Motif-X			F-Motif			MoDL
Order	Motif	Score with (*BH*, *BA*)	Index	Motif	Score with (*BH*, *BA*)	Appearance in (*BH*, *BA*)
1	......SD.E...	(27.45, 27.48)	1	......SD.E...	(31.64, 31.61)	(✓, ?)
2	......S..E...	(16.00, 16.00)	*1	......S..EE..	(23.69, 23.68)	(?, ?)
3	......S..D...	(16.00, 16.00)	3	......S..D...	(15.80, 15.70)	(?, ?)
4	......SQ.....	(16.00, 16.00)	4	......SQ.....	(16.00, 16.00)	(?, ✓)
5	......SP.....	(7.61, 7.36)	2	......S..E...	(15.84, 15.85)	(?, ?)
6	...R..S......	(7.95, 7.99)	5	......SP.....	(7.61, 7.36)	(?, ?)
			6	...R..S......	(7.95, 7.99)	(?, ?)

The first three columns correspond to the output generated by Motif-X while the next three columns correspond to F-Motif. The last column uses symbols “✓” and “?” to indicate the appearance of a motif identified by MoDL or not, respectively. The entry (*1) in row 2 of the right half of the table shows a novel motif that Motif-X could not discover. This novel motif “……S..EE..” also has a very high score (see column 6).

In Table 3, we perform a detailed analysis of the MoDL results. As noted previously, we break the mixture motifs found by MoDL into motifs with single residues. The mixture motifs found by MoDL (with *BH* or *BA*) are shown in column 2 of Table 3, and the corresponding single-residue motifs are separately displayed, in column 3. The columns labeled “Foreground match” and “Background match” show the number of times the associated single-residue motif appears in the foreground and background data, respectively. The last column, “Individual position score”, indicates the motif score for each of the position-residue pair in each motif. Here we can make the following observations on the MoDL results: (a) Apparently, some single-residue motifs found by MoDL are not really good motifs because they appear too infrequently in the foreground data (less than the minimum required frequency of a motif, *M* = 20 for Motif-X and F-Motif); and (b) Aside from these non-significant motifs which fail to satisfy the frequency requirements, mentioned in (a), MoDL is able to find only a few good motifs found by Motif-X and F-Motif (shown in the last column of Table 2, using symbols “✓” and “to indicate the appearance ” to indicate the appearance of a motif or not, respectively). Note that, some motifs found by MoDL, e.g., “……S‥E.D.”, “……S‥E.E.”, and “……SD…‥” in Table 3, satisfy the frequency requirements, but they do not appear in Table 2. Actually, those motifs appear in our *CML* list (please refer to our F-Motif Web site), but after our Step 3, only the seven motifs shown in Table 2 are selected by F-Motif. Such observations regarding MoDL results can also be made on other experiments. Thus, the MoDL results are not really comparable with Motif-X and F-Motif results. Hence, in this study, we primarily focus on the comparison between Motif-X and F-Motif, and MoDL results are included merely for reference.

**Table 3:** Motifs identified by MoDL using the foreground data set, *FM* and background data sets, *BH* and *BA*.

Data set	Mixture motifs	Motifs with single residues	Foreground match	Background match	Individual position score
*FM*(with *BH*)	......S[DEGQ].[DE]...	......SD.D...	13	998	15.64; 12.52
		......SD.E...	33	1536	15.64; 16.00
		......SE.D...	10	1003	4.66; 12.52
		......SE.E...	19	2046	4.66; 16.00
		......SG.D...	9	704	0.47; 12.52
		......SG.E...	5	1025	0.47; 16.00
		......SQ.D...	2	548	13.04; 12.52
		......SQ.E...	9	873	13.04; 16.00
	......S..[ADES].[DE].	......S..A.D.	1	693	0.08; 9.80
		......S..A.E.	4	998	0.08; 11.40
		......S..D.D.	10	968	12.52; 9.80
		......S..D.E.	9	1188	12.52; 11.40
		......S..E.D.	22	1167	16.00; 9.80
		......S..E.E.	32	2184	16.00; 11.40
		......S..S.D.	2	1477	0.24; 9.80
		......S..S.E.	6	1868	0.24; 11.40
*FM*(with *BA*)	......S..[DE].[DE].	......S..D.D.	10	1437	12.42; 9.73
		......S..D.E.	9	1762	12.42; 11.55
		......S..E.D.	22	1756	16.00; 9.73
		......S..E.E.	32	3196	16.00; 11.55
	...…S[DQ].....	......SD.....	54	17260	15.61
		......SQ.....	49	16753	13.05

In columns 2 and 3, the mixture motifs found by MoDL (with *BH* or *BA*) and the corresponding motifs with single residues are displayed, respectively. Columns “Foreground match” and “Background match” show the number of times the associated single residue motif appears in the foreground and background data, respectively. “Individual position score” column indicates the motif score for each of the associations between the position-residue pair in each motif.

#### Experiment 2: *FA* in conjunction with *BA* or *FH* in conjunction with *BA* and *BH*

Two sets of simulations are performed in this experiment (using both foreground and background data collected from the Phospho.ELM database): one uses the foreground data set considering all species (*FA*) in conjunction with the background data set, *BA*; another uses the foreground data set considering only human species (*FH*) in conjunction with both background data sets, *BH* and *BA*. As we have stated earlier, for all experiments we check whether the *P*-value at each residue/position pair is less than 10^−6^. For this experiment, as an illustration, we show that relaxing the constraint on *P*-value results in several additional motifs with high scores, which are indicated using a pair of parentheses in the index column in the associated tables.

Table 4 depicts the motifs found by both algorithms (Motif-X and F-Motif) when we use kinase specific foreground data considering all species (*FA*) and the background data considering all species (*BA*). In these experiments, as shown in Table 4 and in other related tables we use just one column (column 2) to show the motifs found by F-Motif. If a motif is found only by our method (and not by Motif-X) then that motif is marked with an asterisk in column 2 (We have summarized the motifs found only by Motif-X but not by F-Motif in another table). The information in other columns of the table corresponds to F-Motif. The column labeled “Match/Total” shows the number of times the associated motif appears in the present (remaining) foreground data. For example, in the first row, the value 98/306 indicates that the motif “…RR.S……” appears 98 times in the foreground data set of size 306. The next column, PCM hit frequency, gives the number of times, out of the fifty iterations, the associated motif is detected and it refers to the PCM encoding. For example, in the first row the value 37/50 indicates that the motif is generated 37 times in 50 iterations. The sixth column, Background match, displays the percentage of the present (remaining) background data that has matched with the associated motif. Note that, if we do not remove the repeated sequences, F-Motif can find some new motifs satisfying the criteria on statistical significance. These motifs are listed using a “$” symbol in column 2. For such motifs, we do not list values for columns 4 to 7 because these values depend on the order in which the associated motif is detected while using the data without removing the repetitions. We observe that for PKA foreground data there is one novel motif “.R.R‥S……” found by F-Motif when the repeated sequences are not removed.

**Table 4:** Motifs identified by F-Motif and Motif-X using the kinase specific all species foreground data sets (*FA_PKA_*, *FA_PKC_*, *FA_CK2_*, *FA_CDK_*) and the all species background data set (*BA*) with *G* = 15 and *T* = 15.

Data set	Index	Motif	Match/Total	PCM hit frequency	Background match	Motif score
*FA_PKA_*	1	...RR.S......	98/306	37/50	0.59%	32.00
	2	...RK.S......	36/208	50/50	0.41%	32.00
	3	....R.S......	76/172	50/50	4.94%	16.00
	4	...R‥S......	52/96	50/50	5.75%	16.00
	$1	.R.R‥S......				
*FA_PKC_*	*1	...RR.S......	22/297	2/50	0.59%	32.00
	1	......S.R....	77/275	50/50	5.15%	16.00
	3	......S.K....	54/198	50/50	5.58%	16.00
	2	...R‥S......	45/144	50/50	5.78%	16.00
	4	....R.S......	32/99	50/50	5.11%	16.00
	$1	......S.RR...				
	$2	...K‥S.K....				
*FA_CK2_*	1	......SD.E...	36/241	11/50	0.65%	32.00
	*1	......S.EE...	22/205	19/50	0.92%	31.60
	3	......SD.D...	23/183	22/50	0.44%	27.91
	2	......S‥E...	55/160	50/50	6.62%	16.00
	4	......S‥D...	42/105	50/50	5.44%	16.00
*FA_CDK_*	1	......SP.K...	44/209	35/50	0.45%	30.62
	*1	......SP.R...	27/165	22/50	0.53%	22.69
	*(2)	......SP.P...	24/138	8/50	0.99%	21.44
	*(3)	......SP.S...	21/114	24/50	1.01%	17.43
	2	......SP...‥	84/93	33/50	5.51%	16.00

An asterisk in column 2 indicates a new motif that is found by F-Motif but not found by Motif-X. The information in other columns corresponds to F-Motif. The fourth column labeled “Match/Total” shows the number of times the associated motif appears in the present (remaining) foreground data. The fifth column, PCM hit frequency, gives the number of times, out of the fifty iterations, the associated motif is detected and it refers to the PCM encoding. The sixth column, Background match, displays the percentage of the present (remaining) background data that has matched with the associated motif. Also a “$” symbol in column 2 indicates a new motif (satisfying the criteria on statistical significance) that is found by F-Motif but not found by Motif-X if we do not remove the repeated sequences.

From Table 4, we find that for PKC foreground data, out of five motifs found by our method, the top motif (both in terms of Motif score and number of conserved positions) is not identified by Motif-X. If we allow the repeated sequences, F-Motif can discover two new motifs each satisfying the criterion on *P*-value. Similarly, for the CK2 and CDK kinases our method could find 1 and 3 novel motifs, respectively. For CDK kinase, two novel motifs do not satisfy the constraint on P-value. These motifs are indicated by parentheses in the index column. It is also interesting to note that for the PKA and PKC kinases all motifs identified by Motif-X are detected by F-Motif in almost all 50 iterations.

Some other interesting observations from Table 4 are:

- For PKA kinase, the conserved sites appear only on the *left* side of S.
- For CK2 and CDK kinases, the conserved sites appear only on the *right* side of S.
- For the CDK kinase, for all motifs, the position adjacent to S on the right side is conserved with P, making this a strong signature for CDK kinase.

In the second set of simulations we consider kinase specific human species foreground data (*FH_PKA_*, *FH_PKC_*, *FH_CK2_*, *FH_CDK_*) in conjunction with two kinds of background data, only human species (*BH*) and all species (*BA*). Table 5 is divided into 4 groups, each corresponding to a different foreground data set as indicated in the first column of the table. For example, the first group shows the 4 motifs found by Motif-X and F-Motif using the foreground-background pair, (*FH_PKA_*, *BH*) and also the foreground-background pair, (*FH_PKA_*, *BA*). In this case, the change of background has not changed the motifs found by either algorithm. This is true for all other kinases. Note that, except for the CDK kinase, for both methods we get the same set of motifs and all of them satisfy the constraint on the *P*-value, i.e., the position/residue pair is statistically significant. For CDK kinase, F-Motif finds a novel motif, but for this motif the *P*-value at every site is not lower than the threshold for both *BA* and *BH* (again indicated by parentheses in the index column).

**Table 5:** Motifs identified by F-Motif and Motif-X using the kinase specific human species foreground data sets (*FH_PKA_*, *FH_PKC_*, *FH_CK2_*, *FH_CDK_*) and the human background and all species background data sets (*BH*, *BA*) with *G* = 15 and *T* = 15.

Data set	Index	Motif	Match/Total	PCM hit frequency	Background match	Motif score
*FH_PKA_*	1	...RR.S......	58/187	(50, 48)/50	(0.58, 0.59)%	(32.00, 32.00)
	2	...RK.S......	26/129	(50, 50)/50	(0.43, 0.41)%	(31.58, 31.92)
	3	....R.S......	45/103	(50, 47)/50	(4.91, 4.94)%	(16.00, 16.00)
	4	...R‥S......	30/58	(50, 50)/50	(5.75, 5.75)%	(16.00, 16.00)
*FH_PKC_*	1	......S.R....	62/209	(50, 50)/50	(5.19, 5.17)%	(16.00, 16.00)
	2	...R‥S......	46/147	(50, 50)/50	(6.29, 6.31)%	(16.00, 16.00)
	3	......S.K....	37/101	(50, 50)/50	(5.79, 5.60)%	(16.00, 16.00)
	4	....R.S......	20/64	(50, 50)/50	(5.07, 5.11)%	(10.55, 10.49)
*FH_CK2_*	1	......SD.E...	27/177	(6, 2)/50	(0.66, 0.65)%	(32.00, 32.00)
	2	......S‥E...	56/150	(50, 50)/50	(7.45, 7.44)%	(16.00, 16.00)
	3	......S‥D...	53/94	(49, 47)/50	(5.85, 5.88)%	(16.00, 16.00)
*FH_CDK_*	1	......SP.K...	34/155	(31, 34)/50	(0.44, 0.45)%	(27.93, 28.36)
	*(1)	....P.SP...‥	23/121	(28, 24)/50	(0.97, 1.02)%	(21.73, 21.53)
	2	......SP...‥	89/98	(44, 49)/50	(6.78, 6.93)%	(16.00, 16.00)

An asterisk in column 2 indicates a new motif that is found by F-Motif but not found by Motif-X. The information in other columns corresponds to F-Motif. The fourth column labeled “Match/Total” shows the number of times the associated motif appears in the present (remaining) foreground data. The fifth column, PCM hit frequency, gives the number of times, out of the fifty iterations, the associated motif is detected and it refers to the PCM encoding. The sixth column, Background match, displays the percentage of the present (remaining) background data that has matched with the associated motif.

Some of the observations made regarding Table 4 are also valid for Table 5. For example, for PKA kinase, the positions on the *left* side of S are conserved while for CK2 kinase, only sites at the *right* of S are conserved. Like Table 4, for all three motifs for CDK kinase, P is conserved immediately on the right of S.

In Tables S1 and S2, we also show the MoDL results for *FA* in conjunction with *BA*, and *FH* in conjunction with *BH*, respectively (the results for *FH* in conjunction with *BA* are not shown in the tables since the list of motifs is the same as that by *FH* in conjunction with *BH*). Here for four kinase specific groups, the same two observations made on Table 3 for the MoDL results also exist. Further those motifs found by MoDL which satisfy the frequency requirements are still in our *CML* list for this experiment (please refer to our F-Motif Web site).

#### Experiment 3: *FS* in conjunction with (*BH* plus *FS*)

In this experiment, we further validate our approach using the synthetic foreground data set, *FS*, used by Schwartz and Gygi [16]. The background data set for this experiment is obtained by combining the data set, *BH*, and the foreground data set, *FS*, to ensure that the combined background data set also includes the synthetic peptides from the foreground data set. As mentioned in the Materials and Methods, this foreground data set was artificially generated by Schwartz and Gygi [16] and contains five specially designed synthetic motifs. Table S3 shows 28 motifs found by F-Motif. Of these 28 motifs, 9 motifs (including two of the five designed motifs, “….R.S‥L…” and “…‥KS…I‥”) are found both by F-Motif and Motif-X, and the remaining 19 novel motifs are *not* found by Motif-X. Since Motif-X finds a total of 12 motifs, including the other three artificially designed motifs, “….R.S‥P…”, “…TV.S.E….”, and “…D‥SQ.N…”, we perform the following experiment to examine why out of all the motifs, F-Motif could not find those three artificially designed motifs. As shown in Table S4, we use the list of motifs found by Motif-X and further check whether the *P*-value is smaller than 10^−6^ (e.g., motif score ≥6) for each residue/position pair in each motif (here, after a motif is considered, we remove the occurrence of that motif from the foreground and background data sets as this is the philosophy used by Motif-X). Surprisingly, these three artificially designed motifs (marked by symbol # in Table S4) not found by F-Motif do not satisfy the constraint on *P*-value that must be satisfied by the computational definition of motifs used by both F-Motif and Motif-X. This then explains why F-Motif did not find them, but raises the question as to why/how Motif-X *could* find them! Although we do not know for sure the reason behind these results, we think there might be some issues relating to computation of the motif score performed by Motif-X. The overall results demonstrate the consistency and stability of F-Motif, and its ability to discern subtle or delicate patterns. We again emphasize that there is no reason to say these new motifs are false positive because they are patterns that satisfy the definition of motifs.

We have also run MoDL on this data set. As shown in Table S5, MoDL cannot find any of the artificially designed motifs. Furthermore, one motif identified by MoDL (“……S‥P…”) does not satisfy the constraint on *P*-value.

#### Experiment 4: *FMS* in conjunction with *BM*

Now we consider the mouse mass spectrometry data [22] as the foreground data set in conjunction with the background data set, IPI mouse data. Here we randomly use half of peptides extracted from IPI mouse data as the background data set because the Motif-X program is unable to use too large a data set as the input. To justify the use of the randomly selected half of the data, we use the Chi-Square test to check whether there exists a statistically significant difference in distributions of residues in each of the 13 peptide positions between using all of the data and half of the data. In Table S9 (in excel file) each column represents one of the 13 peptide positions, the 30 rows represent 30 simulations, and each cell represents results for one simulation for each peptide position using two data sets (i.e., all of the data and only the randomly selected half of the data). The number in each cell represents the Chi-Square statistic. Since there are 20 amino acids used for the Chi-Square test, we refer to the Chi-Square table with the degree of freedom = 19 to determine whether a statistically significant difference exists or not. The critical value of Chi-Square at level of significance 0.05 is 30.14. In addition to the 30 rows in Table S9, the two additional rows represent the maximum and minimum values of Chi-Square, respectively, for the 30 simulations in each column. In Table S9, the maximum value in each column is less than 30.14 indicating that there is no statistically significant difference between the distributions obtained using the randomly selected 50% of data and the entire data set. Thus the use of the 50% randomly selected background data set is adequate.

Table S6 displays 99 motifs found by F-Motif, of which 25 motifs are found both by F-Motif and Motif-X, and the remaining 74 are novel motifs found by only F-Motif. Moreover, Table S7 shows the 21 motifs found by Motif-X which are *not* identified by F-Motif (and which do not appear on Table S6). Actually, Table S7 is part of our composite motif list *CML* (please refer to our F-Motif Web site), and it shows that except for two of the 21 motifs (indicated by “#”), i.e., “…RT.S……” and “……S…D‥”, all motifs appear in our composite motif list *CML* after 50 iterations. However, instead of 50 iterations, if we use 100 iterations, then these two motifs also appear in *CML*. We also include the MoDL results for this case (see Table S8).

### Robustness with Respect to Encoding of Data

To demonstrate the *numerical stability* of F-Motif with respect to the coding schemes (Binary, PCM, and PWM [28], [29]), we want to use a bigger foreground data set so that a relatively bigger motif set can be identified. For this we use 697 distinct peptides that are obtained combing four different kinase specific human foreground data sets: *FH_PKA_*, *FH_PKC_*, *FH_CK2_*, and *FH_CDK_*. For notational simplicity, we call it *FH_COMBINED_* and use it in conjunction with *BH*. We have repeated the clustering experiments 100 times. The results of these experiments are summarized in Table 6.

**Table 6:** Comparison of motifs discovered by F-Motif using three coding schemes for the human species foreground data set, *FH_COMBINED_*, and the human species background data set (*BH*).

Data set	Motif	Binary hit frequency	PWM hit frequency	PCM hit frequency
*FH_COMBINED_*	...RR.S......	100	100	100
	......SD.E...	39	88	88
	......SP...‥	98	99	99
	...RK.S......	97	87	94
	....R.S......	81	100	100
	...R‥S......	41	100	100
	......S‥D...	75	96	92
	......S‥E...	81	98	94
	......S.R....	79	63	99
	......S.K....	70	29	71
	...K‥S......	70	81	70
Average hit frequency/motif		75.55	85.55	91.55
*CML* size		97	28	28
Execution time		1509.628s	98.090s	90.343s

The process is repeated 100 times with *G* = 15 and *T* = 15 using data represented by three different encoding schemes: PCM, PWM and Binary coding. It reveals that the ultimate performance of the F-Motif is practically independent of the three schemes although computation time and the size of *CML* vary significantly with the choice of encoding schemes.

In column 3 of Table 6, we report the frequencies with which different motifs, which are shown in column 2, have appeared over 100 iterations for binary coding. Similarly, column 4 and column 5 depict the frequencies for PWM and PCM coding schemes, respectively. Two interesting observations can be made from Table 6. First, the list of motifs found for the three different coding schemes are identical. For the PCM coding, each of the 11 motifs on average has appeared 92 times while for PWM and binary coding the average number of times each motif has appeared are 86 and 76, respectively. For the PCM coding, 8 of the 11 motifs have appeared 90 or more times, while for PWM and Binary coding these numbers are 6 and 3, respectively. Second, the size of the *CML* for PCM, PWM, and Binary are 28, 28, and 97, respectively. As expected, since with binary coding the dimension of the data becomes large, it takes much more processing time than the other two coding schemes. In fact, PCM and PWM coding take only about 7% of the time required by binary coding. Also note that PCM takes about 8% less time than that required for PWM. All of these suggest three things: F-Motif is quite stable an algorithm, it is robust with respect to the encoding of features, and of the three coding schemes PCM is the best while Binary is the worst.

## Discussion

Since motif refers to a sequence that is characteristic of a specific biochemical function, here phosphorylation, the relative abundance of a sequence in different proteins is useful information suggesting presence of a motif. Hence, in our F-Motif program we allow the user to determine whether (s)he wants to remove copies of the same sequence that are coming from different proteins (for both foreground and background data).

To make a fair comparison with Motif-X, we adopt Equation (3) and its concomitant removal of peptides satisfying the motif as done by Schwartz and Gygi [16] in Step 3 of our algorithm to get the final list of motifs. However, for the current motif, since the matched peptides in the foreground and background data are removed first before extracting the next motif, the order in which motifs are extracted has an impact on the final list of motifs. For example, suppose the motif “…RR.S……” is selected first, and “….R.S……” is the next motif. Then some peptides that match with the motif “…RR.S……” and are removed, also are members of “….R.S……”, and their removal will affect the frequency of “….R.S……” and influence extraction of subsequent motifs. Hence, in future research, we may use the frequency of a motif in foreground and background data without removing matched peptides to get more motifs.

We have already seen many motifs that are discovered by F-Motif but not by Motif-X with the same foreground and background data sets and using the same protocols. We call these motifs novel. Table 7 summarizes the list of such motifs found in Experiment 2. We shall try to establish that these motifs are indeed novel based on a search of the literature (here we do not make any discussion of the 74 novel motifs found in Experiment 4).

**Table 7:** The list of novel motifs that are discovered by F-Motif but not by Motif-X in Experiment 2.

Foreg./Backg. data sets	Motif	Literature
*(FM, BH, BA)*	......S‥EE‥	**Could not find in the wet-lab literature but in bioinformatics literature ** [**44**] **.**
*(FH_CDK_, BH, BA)*	....P.SP...‥	MAPK substrate motif [33]; probable CDK substrate motif [34].
*(FA_PKA_, BA)*	.R.R‥S......	Akt and PKA substrate motif [35], [36], [37].
*(FA_PKC_, BA)*	...RR.S......	Classical PKA substrate motif [38]; possible PKC substrate motif [39] (also appears in *FA_PKA_*,and *FH_PKA_*).
	......S.RR...	PKC substrate motif [40].
	...K‥S.K....	A possible PKC substrate motif [41].
*(FA_CK2_, BA)*	......S.EE...	Well established CK2 substrate motif [38], [42].
*(FA_CDK_, BA)*	......SP.R...	CDK substrate motif [42].
	......SP.P...	**Could not find in the literature**
	......SP.S...	Phosphorylated by kinases GSK3 and CK1 rather than CDK [43].

The third column refers to literature that has discussed such motifs. There are some novel motifs for which we could not find any clue in the existing literature.

It should be kept in mind that the motifs found by F-Motif might be novel with respect to the motifs found by Motif-X, but these novel motifs might have been well described in the scientific literature consisting of wet-lab experimentations. For example, in Table 7 the motif “….P.SP…‥” found with (*FH_CDK_*, *BH*), (*FH_CDK_*, *BA*) is a classical substrate motif recognized by MAPKs such as ERK1 and ERK2 and subsequently phosphorylated [33]. The F-Motif algorithm categorized this motif under CDK substrate motif possibly because one of the consensus CDK substrate motifs “….PL(S/T)P.(K/R/H)…” [34] contains the “….P.SP…‥” motif. Motif “.R.R‥S……” is an Akt kinase substrate motif [35], [36]. However, it has been observed that in PDE3A the amino acid sequences RRRRSSS which enlists the “.R.R‥S……” motif, is phosphorylated by both PKA and PKB/Akt kinases [37]. “…RR.S……” motif is a classical PKA kinase substrate motif [38]. However, our system has also listed it under PKC. Research revealed that this motif has also been shown to be phosphorylated by PKC. For example, in the NMDA receptor subtype NR2C, the sequences (CTWRRVpSVLES) containing RRXS motif is specifically phosphorylated both by PKA and PKC at the depicted serine site (pS) [39]. Recently it has been shown that PKC phosphorylates four-repeat motifs “……S.RR…” in potassium channel subunit Kir 6.1 and thereby, inhibits the channel function [40]. It is interesting to note that F-Motif algorithm has correctly predicted this “……S.RR…” motif as a probable PKC substrate motif. F-Motif algorithm has also classified “…K‥S.K….” motif as a probable PKC motif. Research reveals that this motif is presented in myelin basic protein (MBP), and that a peptide comprising this motif (Lys-Arg-Gly-Ser^55^-Gly-Lys-Asp) is very well phosphorylated both by PKC and PKA [41].

The CK2 motif “……S.EE…” in Table 7, found in human Hsp-90 and phosphatase inhibitor-2 (EDVGpSDEEE and EQESpSGEED, respectively) are well established for being CK2 substrate [38], [42]. The motif “……SP.R…”, obtained with (*FA_CDK_*, *BA*), is a well recognized CDK substrate motif [42]. The “……SP.S…” motif identified by F-Motif algorithm found to be phosphorylated by other kinases GSK3 and CK1, not included in this study [43]. It remains to be seen whether this motif can also be phosphorylated by CDK. On the other hand, our F-Motif has also identified a good number of peptides containing motifs that have not as yet been found to be associated with any specific kinase. For example, there are no known kinases or interaction domains that specifically target “……S‥EE‥” and “……SP.P…” sequences in wet-laboratory experimental settings. Villén *et al.* [44], have found “……S‥EE‥” motif through their *in silico* phoshorylation motif analysis and mentioned that the motif “……S‥EE‥” is phosphorylated by casein kinase II (CK2). However, no corresponding reference was provided and we could not find any description of this motif in the published literature. A closer look at the motif “……S‥EE‥” reveals that this motif displays an acidic nature, residues C-terminal to the pSer. Further, according to our observations and analysis presented in Tables 4 and 5, it is likely that this motif be phosphorylated by acidic kinase types such as CK2.

### Conclusions

Our algorithm uses exploratory data analysis to discover motifs exploiting statistical information hidden in phosphorylated data. The foreground data is clustered into homogeneous groups, which are analyzed to identify candidate motifs. These candidate motifs are then filtered to find actual motifs with statistically significant motif scores. We use three different encoding schemes including the simplest orthogonal coding and a novel scheme called PCM, which contrasts the foreground data with the background data. Although, the performance of our algorithm practically is independent of the coding schemes, the PCM is found to be the most effective one and orthogonal coding is the least effective one in terms of computation time and consistency. The effectiveness of the algorithm is demonstrated using several data sets and its performance is compared with that of two state-of-the-art methods. In most cases our method could find all statistically significant motifs identified by the other methods. In addition, our method discovered several novel motifs. For some of these additional motifs, we have verified their existence using evidence from the literature. This establishes the excellent motif discovery ability of our algorithm.

## Supporting Information

Table S1 Motifs identified by MoDL using the kinase specific all species foreground data sets (*FA_PKA_*, *FA_PKC_*, *FA_CK2_*, *FA_CDK_*) and the all species background data set (*BA*). (DOC)

Table S2 Motifs identified by MoDL using the kinase specific human species foreground data sets (*FH_PKA_*, *FH_PKC_*, *FH_CK2_*, *FH_CDK_*) and the human background data set (*BH*). (DOC)

Table S3 Motifs identified by F-Motif and Motif-X using the synthetic data set (*FS*) and the combined background data set (*BH* plus *FS*) with *G* = 15 and *T* = 15. (DOC)

Table S4 *P*-value analysis for motifs found by Motif-X using the synthetic data set (*FS*) and the combined background data set (*BH* plus *FS*). (DOC)

Table S5 Motifs identified by MoDL using the synthetic data set (*FS*) and the combined background data set (*BH* plus *FS*). (DOC)

Table S6 Motifs identified by F-Motif and Motif-X using the mouse mass spectrometry data set (*FMS*) and the mouse background data set (*BM*) with *G* = 15 and *T* = 15. (DOC)

Table S7 Motifs identified by Motif-X which are not selected by F-Motif (and which do not appear in Table S6) using the mouse mass spectrometry data set (*FMS*) and the mouse background data set (*BM*). (DOC)

Table S8 Motifs identified by MoDL using the mouse mass spectrometry data set (*FMS*) and the mouse background data set (*BM*). (DOC)

Table S9 Chi-Square test to examine if there is any statistically significant difference in distributions at each of the 13 peptide positions between using half of the data and all of the data for the mouse background data set *BM*). (XLS)

Table S10 (DOC)

Table S11 (XLS)

Text S1 Supplementary text. (DOC)
