# Supplementary material for: Discovery of Protein Phosphorylation Motifs through Exploratory Data Analysis
Source: PLoS One. 2011 May 25;6(5):e20025. doi: 10.1371/journal.pone.0020025 (PMC3102080; doi:10.1371/journal.pone.0020025)
Supplement: Table S3 — (DOC) [file pone.0020025.s003.doc]

**Table S3**.

| Data set | Index | Motif | Match  Total | PCM hit frequency | Background match | Motif score |
| --- | --- | --- | --- | --- | --- | --- |
| *FS* | 1 | ....R.S..L... | 199/9774 | 10 | 0.49% | 32.00 |
|  | *1 | ..L...S...I.. | 42/9575 | 7 | 0.30% | 28.44 |
|  | *2 | ....R.S...I.. | 29/9533 | 10 | 0.17% | 28.31 |
|  | 2 | .....KS...I.. | 137/9504 | 4 | 0.22% | 24.72 |
|  | *3 | ..L.R.S...... | 65/9367 | 2 | 0.42% | 24.66 |
|  | *4 | ..L...S...C.. | 23/9302 | 6 | 0.14% | 20.90 |
|  | *5 | ..L.V.S...... | 60/9279 | 1 | 0.43% | 18.91 |
|  | *6 | ....RES...... | 42/9219 | 2 | 0.25% | 18.43 |
|  | *7 | ..L...S...L.. | 91/9177 | 1 | 0.73% | 16.88 |
|  | *8 | ....VES...... | 40/9086 | 7 | 0.31% | 15.12 |
|  | *9 | ....R.S...F.. | 23/9046 | 4 | 0.13% | 14.81 |
|  | *10 | ..L...S..F... | 22/9023 | 6 | 0.21% | 14.36 |
|  | *11 | ......SQ..L.. | 48/9001 | 2 | 0.39% | 14.17 |
|  | *12 | .....ES..F... | 26/8953 | 5 | 0.15% | 13.86 |
|  | *13 | ..L...S..L... | 75/8927 | 7 | 0.63% | 13.57 |
|  | *14 | ......S..FL.. | 34/8852 | 3 | 0.23% | 12.46 |
|  | 6 | ......S...C.. | 201/8818 | 10 | 1.45% | 9.00 |
|  | *15 | ..L..ES...... | 47/8617 | 7 | 0.34% | 12.90 |
|  | *16 | ....V.S...... | 556/8570 | 2 | 5.22% | 6.71 |
|  | *17 | ..L...S...F.. | 20/8014 | 1 | 0.21% | 12.38 |
|  | *18 | ....R.S...... | 468/7994 | 7 | 4.56% | 7.22 |
|  | 3 | ..L...S...... | 557/7526 | 2 | 5.97% | 6.68 |
|  | *19 | ......S..LF.. | 31/6969 | 8 | 0.25% | 14.08 |
|  | 7 | .....ES...... | 516/6938 | 6 | 5.91% | 6.98 |
|  | 9 | ......S..L... | 615/6422 | 5 | 7.85% | 6.48 |
|  | 8 | ......S..F... | 232/5807 | 4 | 2.84% | 6.47 |
|  | 12 | ......S...F.. | 233/5575 | 6 | 2.98% | 6.42 |
|  | 11 | ......S...L.. | 549/5342 | 1 | 8.31% | 6.61 |
| An asterisk in column 2 indicates a new motif that is found by F-Motif but not found by Motif-X. The information in other columns corresponds to F-Motif. The fourth column labeled "Match/Total" shows the number of times the associated motif appears in the present (remaining) foreground data. The fifth column, PCM hit frequency, gives the number of times, out of the fifty iterations, the associated motif is detected and it refers to the PCM encoding. The sixth column, Background match, displays the percentage of the present (remaining) background data that has matched with the associated motif. | | | | | | |
