# Supplementary material for: Discovery of Protein Phosphorylation Motifs through Exploratory Data Analysis
Source: PLoS One. 2011 May 25;6(5):e20025. doi: 10.1371/journal.pone.0020025 (PMC3102080; doi:10.1371/journal.pone.0020025)
Supplement: Table S4 — (DOC) [file pone.0020025.s004.doc]

**Table S4.**

| Data set | Motif index  in Motif-X | Motif | Motif score  from Motif-X | Individual position score |
| --- | --- | --- | --- | --- |
| *FS* | 1 | ....R.S..L... | 32.00 | 16.00; 16.00 |
|  | 2 | .....KS...I.. | 32.00 | 11.82; 16.00 |
|  | 3 | ..L...S...... | 12.76 | 12.76 |
|  | #4 | ....R.S..P... | 27.08 | 11.08; 4.57 |
|  | #5 | ...TV.S.E.... | 41.55 | 3.91; 9.55; 3.25 |
|  | 6 | ......S...C.. | 9.41 | 9.41 |
|  | 7 | .....ES...... | 9.00 | 9.00 |
|  | 8 | ......S..F... | 7.37 | 7.37 |
|  | 9 | ......S..L... | 8.08 | 8.08 |
|  | #10 | ...D..SQ.N... | 41.08 | 3.08; 9.07; 6.52 |
|  | 11 | ......S...L.. | 6.86 | 6.86 |
|  | 12 | ......S...F.. | 7.03 | 7.03 |
| The symbol "#" indicates those motifs are not found by F-Motif. | | | | |
