# Supplementary material for: Discovery of Protein Phosphorylation Motifs through Exploratory Data Analysis
Source: PLoS One. 2011 May 25;6(5):e20025. doi: 10.1371/journal.pone.0020025 (PMC3102080; doi:10.1371/journal.pone.0020025)
Supplement: Table S7 — (DOC) [file pone.0020025.s007.doc]

**Table S7.**

| Data set | Motif index  in Motif-X | Motif |
| --- | --- | --- |
| *FMS* | 2 | ....P.SP..... |
|  | 5 | ......SP...R. |
|  | 8 | ......SP.R... |
|  | 10 | ...G..SP..... |
|  | 11 | .R..S.S...... |
|  | 13 | ...RR.S...... |
|  | 14 | ......SD.E.E. |
|  | 15 | ......SP.P... |
|  | 18 | ....S.S..DL.. |
|  | 19 | .....DS..E... |
|  | 25 | ......SD.D... |
|  | 27 | ...RK.S...... |
|  | 29 | ....GGS..G... |
|  | #31 | ...RT.S...... |
|  | 36 | R..S..S...... |
|  | 38 | .....GS...... |
|  | 39 | ......SE..E.. |
|  | 40 | ....R.S...... |
|  | #42 | ......S...D.. |
|  | 43 | ......S.P.... |
|  | 44 | ...D..S...... |
| The symbol "#" indicates motifs, which do not appear in our composite motif list *CML* after 50 iterations. | | |
