# Supplementary material for: Discovery of Protein Phosphorylation Motifs through Exploratory Data Analysis
Source: PLoS One. 2011 May 25;6(5):e20025. doi: 10.1371/journal.pone.0020025 (PMC3102080; doi:10.1371/journal.pone.0020025)
Supplement: Table S8 — (DOC) [file pone.0020025.s008.doc]

**Table S8.**

| Data set | Mixture motifs | Motifs with single residues | Foreground match | Background match | Individual position score |
| --- | --- | --- | --- | --- | --- |
| *FMS* | ......S[DP]..... | ......SD..... | 566 | 22773 | 16.00 |
|  |  | ......SP..... | 1146 | 37151 | 16.00 |
|  | ...[DEGKR]..S..[DEPS]... | ...D..S..D... | 45 | 1338 | 12.11; 16.00 |
|  |  | ...D..S..E... | 101 | 1907 | 12.11; 16.00 |
|  |  | ...D..S..P... | 20 | 1424 | 12.11; 8.21 |
|  |  | ...D..S..S... | 26 | 2593 | 12.11; 1.46 |
|  |  | ...E..S..D... | 51 | 1750 | 4.25; 16.00 |
|  |  | ...E..S..E... | 106 | 2903 | 4.25; 16.00 |
|  |  | ...E..S..P... | 19 | 1983 | 4.25; 8.21 |
|  |  | ...E..S..S... | 37 | 3123 | 4.25; 1.46 |
|  |  | ...G..S..D... | 31 | 1571 | 4.99; 16.00 |
|  |  | ...G..S..E... | 60 | 2123 | 4.99; 16.00 |
|  |  | ...G..S..P... | 20 | 2297 | 4.99; 8.21 |
|  |  | ...G..S..S... | 36 | 3732 | 4.99; 1.46 |
|  |  | ...K..S..D... | 32 | 1348 | 4.25; 16.00 |
|  |  | ...K..S..E... | 43 | 2033 | 4.25; 16.00 |
|  |  | ...K..S..P... | 37 | 1651 | 4.25; 8.21 |
|  |  | ...K..S..S... | 38 | 2919 | 4.25; 1.46 |
|  |  | ...R..S..D... | 84 | 1374 | 16.00; 16.00 |
|  |  | ...R..S..E... | 100 | 2076 | 16.00; 16.00 |
|  |  | ...R..S..P... | 125 | 2211 | 16.00; 8.21 |
|  |  | ...R..S..S... | 137 | 3437 | 16.00; 1.46 |
| Columns 2 and 3 display the mixture motifs found by MoDL (with *BM*) and the corresponding motifs with single residues, respectively. Columns "Foreground match" and "Background match" show the number of times the associated single residue motif appears in the foreground and background data, respectively. "Individual position score" column indicates the motif score for each of the position-residue associations in each motif. | | | | | |
