# Supplementary material for: Discovery of Protein Phosphorylation Motifs through Exploratory Data Analysis
Source: PLoS One. 2011 May 25;6(5):e20025. doi: 10.1371/journal.pone.0020025 (PMC3102080; doi:10.1371/journal.pone.0020025)
Supplement: Table S10 — (DOC) [file pone.0020025.s010.doc]

| **Table S10.** | | | | | | | | | | |
| --- | --- | --- | --- | --- | --- | --- | --- | --- | --- | --- |
| Data set | Motif |  | *G*=10 |  |  | *G*=15 |  |  | *G*=20 |  |
|  |  | *T*=10 | *T*=15 | *T*=20 | *T*=10 | *T*=15 | *T*=20 | *T*=10 | *T*=15 | *T*=20 |
| *FH_PKA_* | ...RR.S...... | (***,***) | (***,***) | (***,***) | (***,***) | (***,***) | (***,***) | (***,***) | (***,***) | (***,***) |
|  | ...RK.S...... | (***,***) | (***,***) | (***,***) | (***,***) | (***,***) | (***,***) | (***,***) | (***,***) | (***,***) |
|  | ....R.S...... | (***,***) | (***,***) | (***,***) | (***,***) | (***,***) | (***,***) | (***,***) | (***,***) | (***,***) |
|  | ...R..S...... | (***,***) | (***,***) | (***,***) | (***,***) | (***,***) | (***,***) | (***,***) | (***,***) | (***,***) |
| *FH_PKC_* | ......S.R.... | (***,***) | (***,***) | (***,***) | (***,***) | (***,***) | (***,***) | (***,***) | (***,***) | (***,***) |
|  | ...R..S...... | (***,***) | (***,***) | (***,***) | (***,***) | (***,***) | (***,***) | (***,***) | (***,***) | (***,***) |
|  | ......S.K.... | (***,***) | (***,***) | (***,***) | (***,***) | (***,***) | (***,***) | (***,***) | (***,***) | (***,***) |
|  | ....R.S...... | (***,***) | (***,***) | (***,***) | (***,***) | (***,***) | (***,***) | (***,***) | (***,***) | (***,***) |
| *FH_CK2_* | ......SD.E... | (*,*) | (*,*) | (***,***) | (*,X) | (*,*) | (**,***) | (X,X) | (*,**,) | (***,***) |
|  | ......S..E... | (***,***) | (***,***) | (***,***) | (***,***) | (***,***) | (***,***) | (***,***) | (***,***) | (***,***) |
|  | ......S..D... | (***,***) | (***,***) | (***,***) | (***,***) | (***,***) | (***,***) | (***,***) | (***,***) | (***,***) |
|  | ......S.EE... | (X,X) | (X,X) | (X,X) | (X,*) | (X,X) | (X,X) | (X,*) | (X,X) | (X,X) |
|  | ......S..E.E. | (X,X) | (X,X) | (X,X) | (X,X) | (X,X) | (X,X) | (*,X) | (X,X) | (X,X) |
| *FH_CDK_* | ......SP.K... | (***,***) | (***,***) | (***,***) | (**,***) | (***,***) | (***,***) | (***,***) | (***,***) | (***,***) |
|  | ......SP..... | (***,***) | (***,***) | (***,***) | (***,***) | (***,***) | (***,***) | (***,***) | (***,***) | (***,***) |
| We have experimented with three choices of *G* along with three different choices of *T*. The process is repeated 50 times. Here "***" indicates that the frequency is > 10; "**" indicates that the frequency is in the range of 5 ~ 10; "*" represents a situation when the frequency is in the range of 1 ~ 4; while "X" indicates a frequency of zero. | | | | | | | | | | |
