# Supplementary material for: Discovery of Protein Phosphorylation Motifs through Exploratory Data Analysis
Source: PLoS One. 2011 May 25;6(5):e20025. doi: 10.1371/journal.pone.0020025 (PMC3102080; doi:10.1371/journal.pone.0020025)
Supplement: Text S1 — (DOC) [file pone.0020025.s012.doc]

**Brief Introduction of Motif-X and MoDL**

Motif-X identifies motifs with statistically significant association between residue-position pairs according to a binomially distributed model [16] using a two-step algorithm, which performs a motif-building step followed by a data reduction step. During the motif-building step, the algorithm, using a greedy recursive search, identifies motifs with statistically significant residue/position pairs, and in the data reduction step, all peptides (sequences of a fixed length, in the case of Schwartz and Gygi [16] sequences of length 13) containing the motif just identified in the first step are removed. The whole process is repeated until it fails to return a motif in the first step for want of statistically significant residue/position pairs. The resulting Motif-X score then is the sum of negative logarithm of the *P*-value for each conserved position as defined in (3). Note that, when Motif-X discovers a motif, it deletes all peptides which contain that motif, thereby thinning the dataset. As a consequence of this pruning operation, the score of a motif subsequently found could be artificially low if some of its instances were removed earlier. In our Discussion, we also explain how this pruning process influences the order in which motifs are extracted and how this could impact the final list of motifs. This algorithm uses thresholds on two parameters (*M* and *P*-value) whose choice can/will influence the list of motifs identified. In order to qualify as a motif the *P*-value associated with a position-residue pair should be less than 10^-6^ (i.e., *P* < 10^-6^) and also the motif must occur at least *M* times in the present foreground data set. Schwartz and Gygi [16] used *M* = 20. In our study also, to be consistent with Schwartz and Gygi [16], we have used *M* = 20 for F-Motif.

MoDL uses description length (the amount of information (bits) required to represent the motif set) to produce a set of optimal mixture motifs [17]. The procedure is roughly described as follows. First, an initial set of single-letter motifs (i.e., same type as Motif-X and F-Motif) should be provided in advance as candidate motifs. Second, by performing a greedy iterative approach, a set *W* of potential motif sets at iteration *(i + 1)* are built up from the determined motif set *M(i)* created in the preceding iteration *(i)* by adding or deleting a motif from *M(i)*, or combining two motifs in *M(i)*. Then a motif set *M(i + 1)* ∈ *W* with the minimum description length is chosen for the next iteration. MoDL stops the searching process to arrive at the optimal motif set after executing a prescribed number of iterations or when the description length has not decreased for *n* iterations. Finally, ranking of the individual motifs in the optimal motif set is done by observing the increase in description length when a motif is removed from the set. Note that, to get the motifs with MoDL, in our study no constraint is set in advance and only foreground and background data sets are fed into the MoDL program.

**Foreground and Background Data Sets**

**Foreground data sets:**

*F-All (*"*FA*"*)*: Foreground data sets from the Phospho.ELM database considering all species with respect to PKA, PKC, CK2, and CDK kinase substrates.

Ignoring the species information for proteins (i.e., considering all species together), 1,053 serine-phosphorylated peptides are extracted from Phospho.ELM database for the four kinds of kinases: Protein Kinase A (*FA_PKA_*: PKA, 306 peptides), Protein Kinase C (*FA_PKC_*: PKC, 297 peptides), Casein Kinase II (*FA_CK2_*: CK2, 241 peptides), and Cyclin-dependent Kinase (*FA_CDK_*: CDK, 209 peptides). Here each set of serine-phosphorylated peptides relative to different kinases is utilized as a foreground data set.

*F-Human (*"*FH*"*)*: Foreground data sets from the Phospho.ELM database considering only human species with respect to PKA, PKC, CK2, and CDK kinase substrates.

Considering only human proteins, 728 serine-phosphorylated peptides are extracted from Phospho.ELM database. The set is also divided into the four kinase specific foreground data sets: *FH_PKA_* (PKA, 187 peptides), *FH_PKC_* (PKC, 209 peptides), *FH_CK2_* (CK2, 177 peptides), and *FH_CDK_* (CDK, 155 peptides). The Phospho.ELM database for the four kinds of kinases has 19 species of which Homo sapiens is the most dominant (728 peptides out of 1053).

*F-Multi (*"*FM*"*)*: Foreground data set comprised multiple sets of ATM, Casein II, CaMK II, and MAPK kinase substrates [16].

This foreground data set is directly adopted from the supplementary material of a previous study [16], which contains 298 serine-phosphorylated peptides considering the following four kinds of kinases: Ataxia Telangiectasia Mutated (ATM, 43 peptides), Casein II (184 peptides), Calcium/Calmodulin-dependent protein Kinase II (CaMK II, 41 peptides), and Mitogen-Activated Protein Kinase (MAPK, 30 peptides).

*F-Synthetic (*"*FS*"*)*: Foreground data set consisting of artificially synthetic peptides as used in [16].

This foreground data set is directly adopted from the supplementary material of a previous study [16], which contains 9,774 synthetic peptides with five specially designed synthetic motifs "...D..SQ.N...", "....R.S..L...", "...TV.S.E....", "....R.S..P...", and ".....KS...I..".

*F-MassSpectrometry (*"*FMS*"*)*: Foreground data set from mouse mass spectrometry data [22].

This is a large-scale mass spectrometry data set with 4,189 mouse serine-phosphorylated sites considering phosphorylation probabilities ≥ 0.8 under multiple TiO_2_ conditions [22]. This foreground data set is directly adopted from the supplementary material of a previous study [22]. Note that, only phosphorylated peptides with 13-mers centered at serine are used.

**Background data sets:**

As mentioned in Materials and Methods section, examining all proteins in the Phospho.ELM database, we have extracted peptides with length of 13 centered at serine to generate one kind of background data. In fact, we have extracted *two* background data sets: Human species background data set and Mixed species background data set. Also to analyze mouse mass spectrometry data, we use another background data set from the IPI mouse database (http://www.ebi.ac.uk/IPI/). This data set also considers peptides of length 13 centered at serine from mouse proteins. Note that, similar philosophy is followed by other investigators [16]. Such a background data set consists of both phosphorylated and non-phosphorylated peptides.

*BH (*"*B-Human*"*)*: Human background data set from the Phospho.ELM database.
233,805 peptides are collected for this background data set.

*BA (*"*B-AllSpecies*"*)*: All species background data set from the Phospho.ELM database.
346,248 peptides are gathered for this background data set.

*BM (*"*B-Mouse*"*)*: Mouse background data set from the IPI mouse database.

For this, first 1,029,584 peptides are assembled and then half of them are randomly selected (514,792 peptides) and used as the background. This is explained in the Results section.

**K-means Clustering Algorithm**

Given a data set $X=\left\{\mathbf{x}_{1},\text{ }\mathbf{x}_{2},\text{ }\cdots,\text{ }\mathbf{x}_{n}\right\}$, $\mathbf{x}_{i}\in R^{p}$ and the number of clusters *k*, the *k*-means algorithm runs as follows:

**Step 1**: Randomly select *k* distinct data points from $X$ as the initial prototypes, $V^{0}=\left\{\mathbf{v}_{1}^{0},\text{ }\mathbf{v}_{2}^{0},\text{ }\cdots,\text{ }\mathbf{v}_{k}^{0}\right\}$, $\mathbf{v}_{i}^{0}\in R^{p}$. $\mathbf{v}_{i}$ is the representative (cluster prototype) of the *i*^th^ cluster. Set *t* = 0.

**Step 2**: Assign every data point $\mathbf{x}_{i}$; $i=1,\text{ }2,\text{ }\cdots,\text{ }n$, to the cluster *c* when $\mathbf{x}_{i}$ is closest to the *c*^th^ cluster center $\mathbf{v}_{c}^{t}$.

**Step 3**: Compute the new cluster center, $\mathbf{v}_{i}^{t+1}$ for the *i*^th^ cluster, $i=1,\text{ }2,\text{ }\cdots,\text{ }k$, as the mean vector of the data points assigned to the *i*^th^ cluster; $V^{t+1}=\left\{\mathbf{v}_{1}^{t+1},\text{ }\mathbf{v}_{2}^{t+1},\text{ }\cdots,\text{ }\mathbf{v}_{k}^{t+1}\right\}$.

**Step 4**: If$\left\|V^{t+1}-V^{t}\right\|$ < a tolerance (say, 10^-5^) then Stop, else *t* = *t* + 1; go to Step 2.

In our experiments, while using the PCM and PWM encoding method, each 13-mers is represented by a real valued vector in 13 dimension. However, if we use binary (orthogonal) encoding, then each 13-mers is represented by a vector in 260 (= 13 x 20) dimension. In binary coding each residue is encoded by a binary vector of length 20. Hypothetically, if we number the 20 residues as 1 to 20, then code for the *i*^th^ residue is a 0-1 vector of length 20 where all but *i*^th^ position is 1. Since the central residue is fixed, it is not necessary to encode it as there will be no effect of the central residue irrespective of whether we encode it or not.

**Effect of choices of *G* and *T***

The F-Motif has two algorithmic parameters, *G* and *T*. In Table S10 and Table S11, as an example, we use Experiment 2 to show the influence of these parameters. Here we have considered *G* = 10, 15 and 20 along with *T* = 10, 15, and 20. In Table S10 and Table S11 we report only the motifs for which the constraint on the *P*-value is satisfied. In Table S10 and Table S11, three asterisks (***) indicate that the corresponding motif appears more than 10 times in 50 iterations, two asterisks (**) indicate a frequency between 5 and 10, a single asterisk corresponds to a frequency of < 5, while X means that the corresponding motif never appears in 50 iterations. Comparing Table 4 and Table 5 with Table S10 and Table S11, respectively, we find that the list of motifs remains virtually the same for all chosen parameters. In some cases, a very few motifs do not appear in any of the 50 iterations or appear only for a few combinations of parameters, for example, the motifs "......S.EE..." and "......S..E.E." (with foreground data *FH_CK2_*). However, for some other data sets, particularly for bigger data sets, there may be noticeable differences in the lists produced by different choices of *G* and *T*. Does it mean that the motifs which are not common over different choices of G and T are poor motifs or false positives? No, we cannot make any such inference! Unless and until, wet-lab verification is done, the motifs which are not common are equally good as the common motifs because all of them satisfy the requirements of our computational definition of Motifs. However, if we can have a reasonably large foreground data set and a reasonably large background data set with “complete” ground truth (meaning that for the foreground data we know all motifs that are present), then using a cross-validation type mechanism we may be able to find “optimal” choices for *G* and *T*.

| **Table S10. Motifs discovered by F-Motif using the kinase specific all species foreground data sets (*FA_PKA_*, *FA_PKC_*, *FA_CK2_*, *FA_CDK_*) and all species background data set (*BA*) for various choices of *G* and *T****.* | | | | | | | | | | |
| --- | --- | --- | --- | --- | --- | --- | --- | --- | --- | --- |
| Data set | Motif | *G*=10 | | | *G*=15 | | | *G*=20 | | |
|  |  | *T*=10 | *T*=15 | *T*=20 | *T*=10 | *T*=15 | *T*=20 | *T*=10 | *T*=15 | *T*=20 |
| *FA_PKA_* | ...RR.S...... | *** | *** | *** | *** | *** | *** | *** | *** | *** |
|  | ...RK.S...... | *** | *** | *** | *** | *** | *** | *** | *** | *** |
|  | ....R.S...... | *** | *** | *** | *** | *** | *** | *** | *** | *** |
|  | ...R..S...... | *** | *** | *** | *** | *** | *** | *** | *** | *** |
| *FA_PKC_* | ...RR.S...... | *** | * | * | *** | * | ** | ** | * | *** |
|  | ......S.R.... | *** | *** | *** | *** | *** | *** | *** | *** | *** |
|  | ......S.K.... | *** | *** | *** | *** | *** | *** | *** | *** | *** |
|  | ...R..S...... | *** | *** | *** | *** | *** | *** | *** | *** | *** |
|  | ....R.S...... | *** | *** | *** | *** | *** | *** | *** | *** | *** |
| *FA_CK2_* | ......SD.E... | *** | * | ** | ** | ** | ** | ** | ** | ** |
|  | ......S.EE... | ** | *** | ** | ** | *** | * | ** | *** | * |
|  | ......SD.D... | *** | *** | ** | *** | *** | ** | *** | *** | ** |
|  | ......S..E... | *** | *** | *** | *** | *** | *** | *** | *** | *** |
|  | ......S..D... | *** | *** | *** | *** | *** | *** | *** | *** | *** |
| *FA_CDK_* | ......SP.K... | ** | *** | *** | ** | *** | *** | * | *** | *** |
|  | ......SP.R... | ** | *** | *** | *** | *** | *** | ** | *** | *** |
|  | ......SP..... | *** | *** | *** | *** | *** | *** | *** | *** | *** |
| We have experimented with three choices of *G* along with three different choices of *T*. The process is repeated 50 times. Here "***" indicates that the frequency is > 10; "**" indicates that the frequency is in the range of 5 ~ 10; "*" represents a situation when the frequency is in the range of 1 ~ 4; while "X" indicates a frequency of zero. | | | | | | | | | | |

| **Table S11. Motifs discovered by F-Motif using the kinase specific human species foreground data (*FH_PKA_*, *FH_PKC_*, *FH_CK2_*, *FH_CDK_*) and the human background and all species background data sets (*BH*, *BA*) for various choices of *G* and *T*.** | | | | | | | | | | |
| --- | --- | --- | --- | --- | --- | --- | --- | --- | --- | --- |
| Data set | Motif |  | *G*=10 |  |  | *G*=15 |  |  | *G*=20 |  |
|  |  | *T*=10 | *T*=15 | *T*=20 | *T*=10 | *T*=15 | *T*=20 | *T*=10 | *T*=15 | *T*=20 |
| *FH_PKA_* | ...RR.S...... | (***,***) | (***,***) | (***,***) | (***,***) | (***,***) | (***,***) | (***,***) | (***,***) | (***,***) |
|  | ...RK.S...... | (***,***) | (***,***) | (***,***) | (***,***) | (***,***) | (***,***) | (***,***) | (***,***) | (***,***) |
|  | ....R.S...... | (***,***) | (***,***) | (***,***) | (***,***) | (***,***) | (***,***) | (***,***) | (***,***) | (***,***) |
|  | ...R..S...... | (***,***) | (***,***) | (***,***) | (***,***) | (***,***) | (***,***) | (***,***) | (***,***) | (***,***) |
| *FH_PKC_* | ......S.R.... | (***,***) | (***,***) | (***,***) | (***,***) | (***,***) | (***,***) | (***,***) | (***,***) | (***,***) |
|  | ...R..S...... | (***,***) | (***,***) | (***,***) | (***,***) | (***,***) | (***,***) | (***,***) | (***,***) | (***,***) |
|  | ......S.K.... | (***,***) | (***,***) | (***,***) | (***,***) | (***,***) | (***,***) | (***,***) | (***,***) | (***,***) |
|  | ....R.S...... | (***,***) | (***,***) | (***,***) | (***,***) | (***,***) | (***,***) | (***,***) | (***,***) | (***,***) |
| *FH_CK2_* | ......SD.E... | (*,*) | (*,*) | (***,***) | (*,X) | (*,*) | (**,***) | (X,X) | (*,**,) | (***,***) |
|  | ......S..E... | (***,***) | (***,***) | (***,***) | (***,***) | (***,***) | (***,***) | (***,***) | (***,***) | (***,***) |
|  | ......S..D... | (***,***) | (***,***) | (***,***) | (***,***) | (***,***) | (***,***) | (***,***) | (***,***) | (***,***) |
|  | ......S.EE... | (X,X) | (X,X) | (X,X) | (X,*) | (X,X) | (X,X) | (X,*) | (X,X) | (X,X) |
|  | ......S..E.E. | (X,X) | (X,X) | (X,X) | (X,X) | (X,X) | (X,X) | (*,X) | (X,X) | (X,X) |
| *FH_CDK_* | ......SP.K... | (***,***) | (***,***) | (***,***) | (**,***) | (***,***) | (***,***) | (***,***) | (***,***) | (***,***) |
|  | ......SP..... | (***,***) | (***,***) | (***,***) | (***,***) | (***,***) | (***,***) | (***,***) | (***,***) | (***,***) |
| We have experimented with three choices of *G* along with three different choices of *T*. The process is repeated 50 times. Here "***" indicates that the frequency is > 10; "**" indicates that the frequency is in the range of 5 ~ 10; "*" represents a situation when the frequency is in the range of 1 ~ 4; while "X" indicates a frequency of zero. | | | | | | | | | | |
